# Association of Door-to-Balloon Delay with Complications and Length of Hospital Stays in Patients with ST-Elevation Myocardial Infarction

**DOI:** 10.3390/jcm15155839

**Published:** 2026-07-26

**Authors:** Mohannad Eid AbuRuz, Fatma Refaat Ahmed, Nizar Alsubahi, Haya Ibrahim Ali Abu Maloh, Ahmad Rajeh Saifan, Mohannad Alkhateeb, Osama Alkouri

**Affiliations:** 1 Basic Nursing Department, Faculty of Nursing, Yarmouk University, Irbid 21163, Jordan; 2 Department of Nursing, College of Health Sciences, University of Sharjah, Sharjah 27272, United Arab Emirates; 3 Critical Care and Emergency Nursing Department, Faculty of Nursing, Alexandria University, Alexandria 21526, Egypt; 4 Department of Health Service and Hospital Administration, Faculty of Economics and Administration, King Abdulaziz University, Jeddah 21589, Saudi Arabia; 5 Department of Health Services Research, Care and Public Health Research Institute (CAPHRI), Faculty of Health, Medicine and Life Sciences, Maastricht University, 6200 MD Maastricht, The Netherlands; 6 Clinical Nursing Department, Faculty of Nursing, Applied Science Private University, Amman 11973, Jordan; 7 School of Public Health and Community Medicine, Institute of Medicine, Sahlgrenska Academy, University of Gothenburg, 40530 Gothenburg, Sweden

**Keywords:** primary percutaneous coronary intervention, reperfusion therapy, acute coronary syndrome, hospital stay duration, adverse cardiovascular outcomes

## Abstract

**Background**: ST-elevation myocardial infarction (STEMI) complications are widespread and have a direct effect on the length of stay (LOS) and mortality of patients. Reperfusion therapy via Percutaneous Coronary Intervention mainly impacts outcomes within the first few hours. Consequently, delaying this procedure is a key concern in STEMI management. The door-to-balloon (D2B) time is a crucial predictor of clinical outcomes. This study investigates whether D2B time can predict post-STEMI complications and length of hospital stay, accounting for sociodemographic and clinical variables. **Methods**: This prospective observational study involved 536 patients with confirmed STEMI, recruited consecutively from 8 hospitals in Amman, Jordan. Patients were classified as delayed if their D2B time was ≥2 h. Logistic regression analyzed the impact of delay on complications, while multiple regression assessed how delay time affected LoS. **Results**: The median D2B time was 108 min (IQR 65–395). The delayed group and patients with a previous AMI had 2.22- and 1.41-times higher risks of complications, respectively, compared to their counterparts. Patients with a history of HTN and DM faced 1.24- and 1.18-times higher risks of complications versus their respective groups. Each additional hour of door-to-balloon delay was associated with an increase of 0.294 days in hospital length of stay. **Conclusions**: D2B ≥ 2 h is linked to increased complications and longer LoS. Timely management can reduce complications, shorten LoS, and lower hospital resource use.

## 1. Introduction

To date, cardiovascular diseases (CVDs) are still the number one cause of death internationally. They are responsible for around one-third of the global deaths annually [1,2]. This is affecting both developed and developing countries simultaneously. Even though CVD mortality has decreased in high-income, developed countries, due to the development in treatment, the worldwide burden is rising. This might be due to advanced age, unhealthy lifestyles, and increasing risk factors such as obesity, diabetes, and hypertension [1,2]. This is more prevalent in low- and middle-income countries, where chronic diseases are becoming more common [1,2].

Among all CVDs, Acute Myocardial Infarction (AMI) is still one of the most dangerous outcomes. If not treated in a timely fashion, AMI might result in high rates of mortality and morbidity. The incidence of AMI remains alarmingly high, with an estimated 18 million new cases diagnosed every year [1,2,3]. In high-income countries, where there is an advancement in the treatment of AMI, such as thrombolysis and Percutaneous Coronary Intervention (PCI), the mortality rates have dropped [1,2,4]. However, the incidence remains significant, especially among older adults and those with comorbidities such as diabetes and hypertension [1,2,5].

This is not the case in low- and middle-income countries, where these risk factors are very high and timely access to medical care is minimal. All these contribute to a higher incidence of AMI in these countries. This necessitates focused prevention and intervention strategies to control undesirable outcomes of AMI, such as complications and mortality [6,7]. These trends highlight the significance of AMI as a critical clinical outcome of CVDs and a central concern for public health initiatives.

Complications after AMI are prevalent and have a direct effect on length of stay (LoS) and patients’ mortality rates. Most prevalent complications include recurrent ischemia, arrhythmias, cardiogenic shock, and heart failure [8,9]. Studies have shown that these complications can double the mortality rates and the LoS in the hospital, especially among those who developed cardiogenic shock after the event [10]. Specifically, ventricular arrhythmias and recurrent ischemia were closely linked to higher rates of in-hospital mortality [8,9].

These rates have been reduced due to advances in management, including pharmacological and interventional procedures such as PCI. Still, these complications remain major contributors to both short- and long-term outcomes post-AMI. Efforts to prevent and promptly manage these complications are essential to improve recovery and reduce the burden on healthcare systems. For about 40 years, PCI has been used with improved outcomes and fewer periprocedural adverse effects [11,12,13]. The most notable effect of reperfusion therapy typically occurs within the first few hours after symptom onset. Therefore, delaying this procedure is the primary concern in treating ST-elevation myocardial infarction (STEMI) [11,14,15].

Door-to-Balloon (D2B) time, which measures the time from a STEMI patient’s first medical contact to initiation of PCI, is a key metric for STEMI treatment. Some studies have identified delays in D2B time as the most significant predictor of clinical outcomes [11,16,17]. While other research has found no impact of D2B time on outcomes, including mortality rates [18,19,20,21,22,23]. A meta-analysis indicated that longer D2B times were associated with poorer outcomes, including increased mortality [24]. However, there was considerable variability among the studies, with approximately half of the studies not demonstrating a correlation between D2B time and worse outcomes or higher mortality rates. Therefore, additional large-sample studies are still warranted to reflect real-world outcomes of contemporary practice in low- to middle-income countries such as Jordan.

## 2. Material and Methods

### 2.1. Study Aim

This study was conducted to determine whether door-to-balloon (D2B) time predicts post-STEMI complications and length of stay after adjusting for sociodemographic and clinical characteristics.

### 2.2. Design, Sample, and Setting

This was a prospective observational study of patients recruited consecutively who met the following inclusion criteria. (1) a confirmed diagnosis of STEMI by a cardiologist, (2) aged 18 years and above, (3) PCI was performed within the first 12 h of diagnosis [25], and (4) signed an informed consent form. Patients were excluded from the study if they had a history of heart failure or cardiogenic shock before STEMI, as these conditions are among the outcomes of interest.

**Figure 1:**
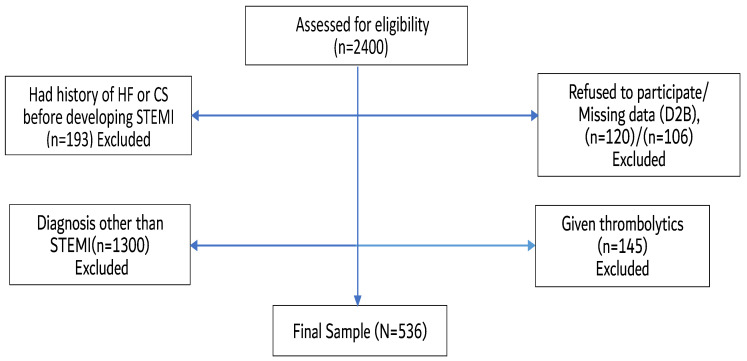
Flow diagram of participants.

Because this study has a dichotomous endpoint (occurrence of complications vs. no complications), an online sample size calculator was used to determine the minimum number of subjects needed to reach adequate power for dichotomous studies [26]. The following criteria were used for this calculation: (1) Alpha of 0.05, (2) power of 0.8, (3) two independent samples; delay (≥2 h), no delay (<2 h), and (4) incidence of complications nearly 7% in the delayed group and 1.99% in the non-delayed group [11]. Based on these criteria, the needed sample size was 534 participants Figure 1.

Patients were recruited from eight hospitals in Jordan: two governmental, one teaching, and five private. These hospitals were selected for their interventional cardiology units and their ability to perform PCI. Additionally, they serve as referral centers for cardiology cases in the region.

### 2.3. Measurement of Variables

Sociodemographic and clinical characteristics: Sociodemographic (age, gender, marital status, and smoking status) and clinical characteristics (history of hypertension, history of diabetes mellitus, history of AMI, history of Coronary Artery Bypass Graft surgery (CABG), and left ventricular ejection fraction (LVEF) were measured either by patient interview or by medical records review.

Delay time: Door-to-balloon (D2B) time was defined as the interval from the patient’s arrival at the PCI-capable hospital (door) to first device activation during primary PCI. As all participating hospitals were PCI-capable centers and no patients were transferred from another healthcare facility prior to PCI, this interval corresponds to the conventional definition of door-to-balloon time [11,23]. Patients were classified in the delayed group if the D2B was ≥2 h (120 min) and in the non-delayed group if the D2B was <2 h [25,27,28]. Although international guidelines recommend a D2B time of <90 min, we used a 120 min threshold to define prolonged D2B as a pragmatic cut-off reflecting local healthcare delivery and logistical constraints in Jordan. This threshold was selected for analytical purposes and should not be interpreted as a guideline-recommended D2B target.

Complications were defined by the occurrence of the following during the hospitalization period and confirmed by a cardiologist: (i) reinfarction; (ii) acute recurrent ischemia; (iii) ventricular fibrillation; (iv) sustained ventricular tachycardia (>15 s) or any ventricular tachycardia necessitating pharmacological and/or electrical intervention; (v) supraventricular tachyarrhythmia with hemodynamic instability; (vi) in-hospital death; (vii) acute pulmonary edema; and (viii) cardiogenic shock [8].

Length of stay: LoS is the number of days the patient remained in the hospital from admission until discharge/death. This data was obtained from medical archives.

### 2.4. Ethical Considerations

This study received official approval from the Applied Science Private University IRB committee in Amman, Jordan (IRB number: Faculty 2023-2024-1-3) and from the participating hospitals. Informed consent was obtained from all eligible participants, including permission to review their medical records. All information was handled with strict confidentiality and securely stored in a protected cabinet at the principal investigator’s office for hard copies and on a password-protected computer for soft copies.

### 2.5. Data Collection Process

A well-trained cardiovascular research assistant (RA) (one per hospital) met with the emergency room head nurses and each shift charge nurse of the selected hospitals and explained the study. The RAs informed the head nurses and charge nurses to call them when they have an admission for a patient with STEMI. To ensure that no patients were missed during the data collection period, each RA reviewed the emergency room admission records at 8 am each day. When a patient is admitted to the hospital, the RA approaches that patient, explains the study, benefits, and risks, and obtains informed consent if the patient agrees to participate. The patients were then asked to complete the sociodemographic sheet once they were hemodynamically stable. All other information, including D2B, clinical data, complications, and LoS, was abstracted from medical records after discharge/death. The interrater reliability among the RAs was 96%. Every RA was asked to extract data from five files, which the Principal Investigator reviewed for consistency.

### 2.6. Data Analysis

Data were analyzed using SPSS version 29 (IBM, Armonk, NY, USA). Continuous variables were presented as mean and standard deviation, while categorical variables were reported as frequencies and percentages. For D2B, a 120 min cut-off was used to define prolonged delays. Differences between groups were investigated using an independent *t*-test or a chi-square test, as appropriate.

Two steps were taken to determine the effect of D2B, sociodemographic, and clinical variables on complications and LoS. First: Correlation between sociodemographic and clinical variables (age, gender, marital status, smoking, history of HTN, history of DM, history of AMI, history of angina, prior CABG, prior PTCA, LVEF, and delay category) and the occurrence of complications and LoS. Two: All variables that showed significant correlations (history of HTN, history of DM, history of AMI, history of angina, prior CABG, prior PTCA, and delay category) were then entered into logistic regression to assess their effect on complications and into multiple regression to assess their impact on LoS. For further clarification, patients who developed any complications were coded as 1 (having any complications), and those with no complications were coded as 0. There was no multicollinearity among the variables, as the VIFs were all less than 2.

## 3. Results

A total of 536 patients with a confirmed diagnosis of STEMI participated in this study, with a mean age of around 62 years. Patients who were delayed for more than two hours were more females, who had diabetes, had lower LVEF, stayed longer in the CCU, and stayed longer in the hospital. Additionally, they had higher numbers of previous AMI, angina, and CABG surgery. Moreover, in this group, the number of patients who developed complications was greater than that of those who did not delay (Table 1). The median D2B time was 108 min [interquartile range (IQR) 65–395]. The independent samples Mann–Whitney test showed a significant difference between patients who delayed (median 460 min, IQR 259–587, *n* = 226) and those who did not delay (median 71 min, IQR 51–96, *n* = 310) (*p* < 0.001).

Acute recurrent ischemia followed by sustained ventricular tachycardia was the most frequent complication. On the other hand, in-hospital death and reinfarction were the least developed complications. It is worth noting that more than one patient developed multiple complications during hospitalization (Table 2).

Delayed group and patients with a history of previous AMI were at 2.22 and 1.41 times higher risk of developing these complications compared to those who did not delay and those who did not have a history of AMI. Additionally, those with a history of DM and those with a history of HTN were at 1.24 and 1.18 times higher risk of developing these complications compared to those who did not have a history of DM and those who did not have a history of HTN (Table 3 and Table 4).

Every 1 h increase in delay was associated with a 0.294-day increase in hospital stay. Similarly, patients with a history of DM and HTN, and those who had previously undergone CABG, increased their LoS in the hospital by 0.103, 0.122, and 0.01 days, respectively. Interestingly, patients who had previously undergone PTCA reduced their hospital LoS by 0.125 days compared with those who had not. The model explained 22.4% of the variance regarding the hospital LoS (Table 5).

## 4. Discussion

The current study provides valuable insights into the impact of door-to-balloon (D2B) time on outcomes in patients with ST-elevation myocardial infarction (STEMI) undergoing percutaneous coronary intervention (PCI) in Jordan, an emerging country. By supporting the notion that longer D2B times can increase the risk of complications and length of stay (LoS), it becomes clear that another piece of the puzzle is in place regarding the key role of the time factor in reperfusion treatment for STEMI in countries with limited reperfusion resources.

Patients who had a D2B time of more than two hours for procedures waited longer for treatment if they were females who had diabetes, with lower LVEF levels, longer CCU and hospital stay times, and more occurrences of AMI, angina, and CABG procedures. Also, there was a higher chance of developing complications for those with D2B times than for those who did not wait for treatment. This can be attributed to the fact that women and diabetics often suffer from atypical or painless symptoms; hence, treatment is often sought longer [29,30,31].

Similarly, those with lower LVEF levels or previous heart-related problems may have complex coronary artery disease that requires further analysis or procedural preparations before treatment can be applied [32]. These factors may explain treatment delays, longer hospitalizations, and higher complication rates in these patient groups.

One of the most significant findings of the current study is the correlation between the occurrence of complications in STEMI patients and the delay in D2B time (≥2 h). Particularly, the findings supported that STEMI patients with delayed D2B times were more likely to present acute ischemia, ventricular tachycardia, and other complications. These results were in line with other studies that have already demonstrated the significant harm associated with the prolonged ischemic time on the myocardium regarding outcomes in STEMI patients [12,17,18]. As an example, the meta-analysis done by Foo et al. in 2018 already found that higher D2B times were linked with higher outcomes, such as higher death rates, in STEMI patients [24]. There could be different reasons for this significance, such as the prolonged ischemic time, which may result in irreversible myocardial damage, cardiac arrhythmia, electrical instability, and subsequent complications. Therefore, several studies and meta-analyses recommended shortening the time of D2B to avoid these complications [12,17,25]. It could also be linked to structural concerns within the hospital system in the region where the research is conducted.

It is worthy to note that the incidence of complications in the current study (27.0% for the whole sample, 32.3% for the delayed group, and 23.2 for no delay) was comparable to those reported in the same country for STEMI (38.4% [33], 37.4% [34], 28.0% [8], and 24.0% [9]). However, they were higher than other international ones. This difference might be due to the effect of the delay time on complications. In the current study, delay time was defined as more than 2 h, whereas in other studies it was measured at 90 min. The actual data reflect this clearly, as the median D2B time was 108 min (IQR 65–395), while it was 54 min (IQR 29–90) in another study [11].

In-hospital mortality was one of the less common adverse outcomes. This was consistent with some previous studies, which reported no impact of D2B on mortality rates [19,20,21,22,23,24]. However, for those with late D2B times and with previous AMI, the risk of mortality was appreciably increased. This supports previous observations that treatment-time-related delays are a strong determinant of increased in-hospital mortality rates due to protracted myocardial ischemic times and increased infarct size [12,17]. Also contributing to infarct-size-related myocardial dysfunction are previous AMI episodes, which further impair myocardial function and reserves to render affected individuals increasingly susceptible to further ischemic damage [24]. Similarly, diabetes and hypertension can be construed to disrupt the infarct vessel integrity further and contribute to the reduced myocardial function, hence further contributing to increased mortality rates [35,36]. However, these results should be interpreted with caution, since in-hospital death accounted for only 10 events (1.9% of the sample), and the increased risk applies to the composite complication endpoint rather than mortality alone.

Additionally, the research showed a significant positive relationship between delayed D2B time and LoS in hospitals. Indeed, research has shown that complications during hospital stays positively affect hospital LoS, as patients with complications have higher LoS [8,9]. Therefore, hospital LoS could be directly attributed to complications in hospitals where patients have delayed D2B times.

Another interesting observation made from the study is the link between existing comorbidities, for instance, diabetes mellitus (DM) and hypertension (HTN), and the risk of complications and long LoS. Specifically, it has been found in the study that for patients with existing DM or HTN, there is a higher probability of complications and longer hospital stays. This observation is supported by evidence from other studies confirming the negative effect of comorbidities on STEMI outcomes [5]. As an illustration, it has been found in the study by Tisminetzky et al. (2021) that for patients with existing chronic conditions like DM and HTN, the probability of incurring complications is higher, together with an extended hospital stay following the patient’s first acute myocardial infarction [5]. The link between existing comorbidities and complications in outcomes could be explained by the propensity on the part of patients with existing comorbidities to be more vulnerable to ischemic injuries consequent on reperfusion following STEMI.

The observation that for patients who had undergone previous PTCA, the LoS is reduced, is noteworthy. There could be many reasons for these observations. First, individuals who have already undergone a PTCA procedure may be more informed about medical processes in general, enabling them to better cope with their recovery and thus leading to earlier hospital discharge. Second, it is possible that these individuals were treated with more aggressive approaches to either their surgery or their subsequent care, thus enabling an early hospital discharge.

Given these results, it could be recommended that in Jordan and other developing countries, the following approaches should be adopted to manage STEMI cases better: First, it is essential to concentrate on shortening the time from diagnosis to treatment (D2B time) by installing optimized protocols for working in collaboration with the EMS system and hospitals [5]. Activation of the cardiac cath lab before hospital admission, in addition to an optimized triage system, could also help shorten the time from diagnosis to treatment in STEMI cases.

Second, targeted interventions should also be done in view of the needs of high-risk groups, for instance, in diabetic, hypertensive, or AMI survivors. In these subsets, more aggressive risk-factor modification, close surveillance for complications, or guideline-directed medical therapy could be considered. Thirdly, there would be an attempt to increase the availability of PCI for eligible STEMI patients, independent of their location or socioeconomic status [6]. There would be a need to establish regional PCI centers and a system for transferring patients for PCI treatment, if needed.

Finally, further research is needed to identify the specific barriers to timely STEMI care in Jordan and to evaluate the effectiveness of various interventions to improve outcomes. This may involve qualitative studies to explore the perspectives of healthcare providers and patients, as well as randomized controlled trials to assess the impact of specific interventions on D2B times, complications, and LoS.

## 5. Limitations

Despite the strengths of this study in understanding the effect of D2B time on STEMI outcomes in Jordan, it is also important to consider the following limitations. First, it is possible that abstraction from the medical records was occasionally erroneous. Second, it is essentially an observational study; hence, its ability to establish cause-and-effect relationships regarding outcomes over the time period in question is limited. Third, the presence of unmeasured factors could affect the relationships reported in these findings. Although multivariable regression analyses were performed, residual confounding cannot be excluded. Variable selection for the multivariable models was based on statistically significant bivariate associations, and several clinically relevant factors, including age, sex, and left ventricular ejection fraction (LVEF), were not retained because they were not significantly associated with the study outcomes in the preliminary analyses. Consequently, the observed associations between door-to-balloon delay, complications, and hospital length of stay should be interpreted with caution. Future studies should employ clinically informed multivariable models that include established confounding variables irrespective of their statistical significance in preliminary analyses.

In addition, hospital-level characteristics (e.g., institutional protocols, staffing patterns, and postoperative care practices) were not collected and therefore could not be included in the analyses. Consequently, the observed associations between D2B delay, postoperative complications, and hospital length of stay should be interpreted with caution, as unmeasured or unadjusted confounders may have influenced the reported estimates. In totality, even with these limitations in mind, it is vital to understand that the research provides significant evidence for early reperfusion therapy in STEMI populations, with respect to reduced time benefits in MEN countries.

## 6. Conclusions

This study demonstrates that prolonged door-to-balloon (D2B) time (≥2 h) in patients with ST-elevation myocardial infarction (STEMI) is associated with a higher risk of in-hospital complications and longer hospital stays, even after adjusting for sociodemographic and clinical characteristics. Patients with comorbidities such as diabetes, hypertension, or a history of AMI are particularly vulnerable to adverse outcomes when treatment is delayed. These findings highlight the critical importance of minimizing hospital delays in reperfusion therapy to improve clinical outcomes, reduce length of stay, and optimize healthcare resource use. The study provides valuable evidence for healthcare providers and policymakers, especially in low- and middle-income countries, emphasizing the need for streamlined STEMI care protocols and targeted interventions for high-risk patient groups.

## Figures and Tables

**Table 1:** Patient characteristics, delayed vs. no-delay group (*n* = 536).

Characteristics	Total Sample (*n* = 536)	Delayed (*n* = 226)	No-Delay (*n* = 310)	t or χ^2^	*p*
Age	61.98 ± 13.5	62.11 ± 13.80	61.88 ± 13.32	0.198	0.84
Gender				3.79	0.032
Male	350 (65.3)	138 (61.1)	212 (68.4)		
Female	186 (34.4)	88 (38.9)	98 (31.6)		
Marital status				2.32	0.82
Married	368 (68.7)	156 (69.6)	212 (68.6)		
Single/Divorced/Widowed	168 (31.3)	70 (30.4)	98 (31.4)		
Current smoker	181 (33.70)	77 (34.7)	104 (33.5)	0.07	0.42
History of HTN	301 (56.2)	134 (53.9)	167 (60.6)	2.4	0.07
History of DM	116 (21.6)	62 (27.9)	54 (17.4)	8.38	0.003
History of AMI	144 (26.9)	72 (32.3)	72 (23.2)	5.41	0.013
History of Angina	182 (34.0)	87 (39.5)	95 (30.9)	4.19	0.025
Prior CABG	45 (8.4)	26 (11.7)	19 (6.1)	5.13	0.018
Prior PTCA	87 (16.2)	46 (20.6)	41 (13.3)	5.13	0.016
Development of complications	145 (27.1)	73 (32.3)	72 (23.2)	5.46	0.013
LVEF	49.8 ± 13.17	47.11 ± 13.28	52.12 ± 12.64	−3.73	<0.001
Hospital LoS in days	5.91 ± 4.49	8.04 ± 5.44	4.40 ± 2.84	9.95	<0.001
CCU LoS in days	3.14 ± 3.07	5.44 ± 4.10	1.83 ± 0.72	15.14	<0.001

Values are either M ± SD or *n* (%); AMI: Acute Myocardial Infarction, CABG: Coronary artery bypass graft, CCU: Critical Care Units, DM: Diabetes Mellitus, HTN: Hypertension, LoS: Length of Stay, LVEF: Left Ventricular Ejection Fraction, PTCA: Percutaneous Coronary Artery Angioplasty.

**Table 2:** Specific complications.

Complication Developed	No. (%) Patients *
Acute recurrent ischemia	108 (20.2)
Sustained ventricular tachycardia	74 (13.8)
Pulmonary edema	70 (13.1)
Supraventricular tachyarrhythmia	41 (7.6)
Ventricular fibrillation	26 (4.9)
Cardiogenic shock	18 (3.4)
Reinfarction	12 (2.2)
In-hospital death	10 (1.9)

* More than one patient has more than one complication.

**Table 3:** Significant correlations between complications, LoS, and delay with sociodemographic characteristics of the sample (*n* = 536).

Character	Delay Category	Hypertension	Diabetes Mellitus	Previous MI	Previous Angina	Prior CABG	Prior PTCA
Complications	0.101 *	0.101 *	0.107 *	0.128 **	0.136 **	0.105 *	0.130 **
Length of Stay	0.567 **	0.094 *	0.144 **	0.110 **	0.148 **	0.150 **	0.099 *

* Correlation is significant at the 0.05 level, ** Correlation is significant at the 0.01 level. CABG: Coronary artery bypass graft, LoS: length of Stay, MI: Myocardial infarction, PTCA: Percutaneous Coronary artery Angioplasty.

**Table 4:** Logistic regression, predictors of complications (*n* = 536).

Predictor	OR	95% CI	Wald	*p* Value
Delay group ≥ 2 h	2.22	1.31–4.22	11.25	0.003
History of previous AMI	1.41	1.22–1.98	11.06	0.005
History of HTN	1.24	1.11–1.48	10.11	0.006
History of DM	1.18	1.06–1.39	9.71	0.007

AMI: Acute Myocardial Infarction, DM: Diabetes Mellitus, HTN: Hypertension.

**Table 5:** Stepwise regression analyses for predictors of hospital LoS (*n* = 536).

Predictor	Unstandardized B	Standardized β	t	95% Confidence Interval	*p* Value	Durbin-Watson Statistics	Model Statistics
Delay time in hours	0.294	0.38	6.96	0.211 to 0.377	<0.001	1.92	R^2^ = 0.224; F_(11,499)_ = 7.75, *p* < 0.001
History of DM	0.103	0.10	2.32	0.016 to 0.190	0.021		
History of HTN	0.122	0.05	2.59	0.030 to 0.214	0.010		
Prior PTCA	−0.125	−0.08	−2.63	−0.246 to −0.004	0.043		
Prior CABG	0.010	0.12	2.19	0.001 to 0.019	0.029		

CABG: Coronary artery bypass graft, DM: Diabetes Mellitus, LoS: length of Stay, PTCA: Percutaneous Coronary artery Angioplasty.

## Data Availability

The datasets generated and analyzed during the current study are available from the corresponding author upon reasonable request.

## Abbreviations

AMI	Acute Myocardial Infarction
CABG	Coronary Artery Bypass Graft
CCU	Coronary Care Unit
CI	Confidence Interval
CVDs	Cardiovascular Diseases
D2B	Door-to-Balloon
DM	Diabetes Mellitus
EMS	Emergency Medical Services
HF	Heart Failure
HTN	Hypertension
IQR	Interquartile Range
IRB	Institutional Review Board
LVEF	Left Ventricular Ejection Fraction
LoS	Length of Stay
MI	Myocardial Infarction
OR	Odds Ratio
PCI	Percutaneous Coronary Intervention
PTCA	Percutaneous Transluminal Coronary Angioplasty
STEMI	ST-Elevation Myocardial Infarction
